# microRNA-21 promotes breast cancer proliferation and metastasis by targeting LZTFL1

**DOI:** 10.1186/s12885-019-5951-3

**Published:** 2019-07-27

**Authors:** Hui Wang, Zheqiong Tan, Hui Hu, Hongzhou Liu, Tangwei Wu, Chao Zheng, Xiuling Wang, Zhenzhao Luo, Jing Wang, Shuiyi Liu, Zhongxin Lu, Jiancheng Tu

**Affiliations:** 1 Department and Program of Clinical Laboratory Medicine, Center for Gene Diagnosis, Zhongnan Hospital of Wuhan University, 169 Donghu road, Wuhan, 430071 People’s Republic of China; 2 Department of Medical Laboratory, The Central Hospital of Wuhan, Tongji Medical College, Huazhong University of Science and Technology, Wuhan, 430014 China; 3 Cancer Research Institute of Wuhan, The Central Hospital of Wuhan, Tongji Medical College, Huazhong University of Science and Technology, Wuhan, 430014 China

**Keywords:** microRNA-21, Breast cancer, Leucine zipper transcription factor-like 1, Epithelial to mesenchymal transition

## Abstract

**Background:**

Breast cancer is the most common cancer type in female. As microRNAs play vital role in breast cancer, this study aimed to explore the molecular mechanism and clinical value of miR-21 in breast cancer.

**Methods:**

qRT-PCR was performed to detect miR-21 levels in plasma of 127 healthy controls, 82 benign breast tumor, 252 breast cancer patients, as well as in breast cancer cell lines. Transwell and wound healing assay were used to analyze breast cancer metastasis in response to miR-21 inhibitor. Colony formation and eFluor™ 670 based flow cytometric analysis were used to test breast cancer proliferation following miR-21 inhibitor treatment. Leucine zipper transcription factor-like 1 (LZTFL1), the target gene of miR-21 was predicted by MIRDB, TargetScan 5.1, PicTar and miRanda. Survival analysis of LZTFL1 levels in breast cancer prognosis was estimated with the Kaplan–Meier method by log-rank test according to data from the Cancer Genome Atlas. Luciferase activity assay was performed to confirm the regulation of miR-21 on LZTFL1. LZTFL1 siRNA and miR-21 inhibitor were co-transfected to breast cancer cells, then cell proliferation, migration and epithelial–mesenchymal transition (EMT) makers were tested. BALB/c nude mice were injected in situ with Hs578T cells stably overexpressing miR-21. Breast tumor growth, metastasis and the expression of EMT markers or LZTFL1 were detected in vivo.

**Results:**

Plasma miR-21 levels were elevated in breast cancer patients compared with healthy controls and benign breast tumor patients, and the miR-21 levels were significantly decreased after surgery comparing with pre operation in 44 patients. Inhibition of miR-21 suppressed cell proliferation and metastasis in breast cancer cells. LZTFL1 was identified as a novel target gene of miR-21. Knockdown of LZTFL1 overcame the suppression of miR-21 inhibitor on cell proliferation, metastasis and the expression of EMT markers in breast cancer cells. miR-21 overexpression promoted breast cancer cell proliferation and metastasis in vivo.

**Conclusions:**

These results indicate that plasma miR-21 level is a crucial biomarker for breast cancer diagnosis and targeting miR-21–LZTFL1–EMT axis might be a promising strategy in breast cancer therapy.

**Trial registration:**

Retrospectively registered.

**Electronic supplementary material:**

The online version of this article (10.1186/s12885-019-5951-3) contains supplementary material, which is available to authorized users.

## Background

Breast cancer is the most common cancer type in female, and many patients are suffered from recurrences and metastasis [1–3]. MicroRNAs (miRNAs) are non-coding, single-stranded RNA molecules that regulate target gene expression via posttranscriptional processing [4, 5]. Recently, several studies indicated the promising role of miRNA in the diagnose and outcome prediction in several cancers [6–12].

miR-21 is upregulated and promotes metastasis in several cancers [13–20]. Our previous study also proved that plasma levels of miR-21 were upregulated in large B-cell lymphoma patients in China [21]. The epithelial–mesenchymal transition (EMT) is a process that epithelial cells lose their cell polarity and cell adhesion ability, which will lead to cancer metastasis [22, 23]. Although miR-21 was indicated to play a crucial role in the metastasis of lung cancer, ovarian cancer and head and neck cancer though several signaling pathways, the molecular mechanism of how miR-21 regulates the EMT process in breast cancer is not clear [24–31].

Leucine zipper transcription factor-like 1 (LZTFL1) is one of the key genes which regulate cancer metastasis [32–35]. Previous study found that LZTFL1, acting as a tumor suppressor, was down-regulated in gastric and lung cancer [34, 35]. Mechanically, LZTFL1 was reported to regulate β-catenin signaling which then activated the EMT in several cancers [35]. In our study, we will explore the new target gene of miR-21 and investigate the mechanism of miR-21 in regulating breast cancer metastasis, in order to provide new insights and strategies for breast cancer therapy.

## Methods

### Patients and plasma samples

The study included 127 healthy control subjects, 82 benign breast cancer patients and 252 first-diagnostic breast cancer patients recruited between 2015 and 2017 from the Central hospital of Wuhan. All breast cancer patients who had undergone surgery without chemotherapy or radiotherapy had been diagnosed by pathological examination. Control subjects were verified to be healthy, based on serum tumor maker analysis, liver function test and chest X-ray. 44 pairs of pre- and post-surgery samplers were matched and compared.

Plasma samples were collected in EDTA tubes and centrifuged at 1000 g at 4 °C for 15 min. Then the supernatants were obtained and stored at − 80 °C until testing. This study was approved by the Ethical and Scientific Committees of the Central Hospital of Wuhan. Clinical data were obtained from the hospital pathologic records.

### Cell lines

The human mammary epithelial cell line HBL-100 (catalogue number: GNHu 10), and human breast cancer cell lines including Hs578T (TCHu127), MDA-MB-231 (TCHu227), SK-BR-3 (TCHu225) and MCF-7 (TCHu74) were obtained from the Cell Bank of the Chinese Academy of Sciences (Shanghai, China) in 2017. HBL-100 cells were cultured in Dulbecco’s Modified Eagle’s medium (DMEM, Hyclone, USA) containing 10% fetal bovine serum (FBS, NQBB, Australia). MDA-MB-231 cells were cultured in Leibovitz’s L-15 medium (Hyclone, USA) containing 10% FBS. Hs578T cells were cultured in DMEM medium with 0.01 mg/ml insulin and 10% FBS. MCF-7 and SK-BR-3 cells were cultured in RPMI-1640 (Hyclone, USA) containing 10% FBS. Cells were cultured at 37 °C in a 5% CO_2_ incubator.

### Plasmids and siRNAs

The miR-21 inhibitor, miR-21 mimic and corresponding negative controls were designed and synthesized by Genepharma (Shanghai, China). LZTFL1 overexpressing plasmid and negative control were purchased from Genechem (Shanghai, China). Small interfering RNAs (siRNAs) for knockdown of LZTFL1 and a negative control were purchased from Genepharma (Shanghai, China). The 3′-UTR of LZTFL1 containing the putative miR-21 recognition elements was amplified from the human genome of Hs578T cells by PCR (sense, 5′- TAT CTA GAC ATT TTG TCA TAT CCC CTC T-3′; antisense, 5′-ATG CGG CCG CAT GTT CAT GTT CAC TGC TGT-3′). The mutated 3′-UTR of LZTFL1 was also amplified (sense, 5′-TAT CTA GAC ATT TTG TCA TAT CCC CTC T-3′; antisense, 5′-ATG CGG CCG CAC ATT GTT GCG CTA CTT AAC ATT TA − 3′). The wild-type and the mutated amplification products were cloned into the downstream of the pRL-TK vector (Promega, USA) between the XbaI and NotI sites. Two constructs were confirmed by DNA sequencing.

### Transfection

Cells were seeded into a 6-well plate at 100,000–300,000 cells/well as described previously [36]. After 12 h of culture, plasmid or siRNA were transfected to cells by using Lipofectamine 2000 (Invitrogen, USA) according to the manufacturer’s protocol. Cells were harvested for analysis after.

### RNA isolation and quantitative RT-PCR

RNAs were extracted from plasma and cell lines using TRIZOL reagent (Invitrogen, USA). RNA was reverse-transcribed using the cDNA Synthesis Kit (Fermentas, Canada). Quantitative real-time PCR (qRT-PCR) was performed with an ABI StepOnePlus™ real-time PCR System (Applied Biosystems, USA) using the SYBR Green mix (Toyobo, Japan) [37]. Glyceraldehyde-3-phosphate dehydrogenase (GAPDH) was used as an internal control for normalization. U6 was used as an internal control for miRNA expression. The relative gene expression were calculated using the 2^-△△Ct^ method.

### Luciferase activity assay

HEK-293 T cells were seeded into 96-well plates (5 × 10^3^ cells/well) before transfection. Then 100 ng of pRL-TK–LZTFL1–3’UTR or pRL-TK–LZTFL1–3’UTR mutant and 10 ng of the pGL3 control (Promega, USA) were co-transfected into cells, along with 60 ng of pSIF–GFP–miR-21 precursor plasmid or 10 pmol of miR-21 inhibitor. After 48 h, luciferase activity was detected using the Dual-Glo luciferase reporter assay system (Promega).

### Western blotting

RIPA lysis buffer (Beyotime, Shanghai) was used to obtain total protein from cells. Protien concentrations were measured using a BCA assay kit (Beyotime, Shanghai). Protease inhibitors (Sigma-Aldrich, USA) were supplemented to the cellular extracts. Then proteins were separated by sodium dodecyl sulfate (SDS) polyacrylamide gel electrophoresis (PAGE), and transferred onto a 0.45-μm PVDF membrane (Millipore, USA) at 200 mA for 90 min on ice. After blocking with 5% fat-free milk for 2 h at room temperature, the PVDF membranes were incubated with primary antibodies (Cell Signaling Technology, USA) at 4 °C overnight. Horseradish peroxidase (HPR)-conjugated secondary antibodies (Cell Signaling Technology, USA) were used to bind the primary antibodies. ChemiDoc XRS+ system with Image Lab software (Bio-Rad, CA, USA) was used for visualization. Protein expression was quantified by using Image J software (National Institutes of Health, Bethesda, Maryland).β-actin was used as a control for normalization.

### Colony formation assay

Cells were seeded in six-well plates in triplicate at densities of 1 × 10^3^ per well and were treated with miR-21 inhibitor and its corresponding control. After 14 days, cells were washed with 1 × PBS, and fixed in methanol for 15 min at room temperature. Then cells were stained with crystal violet for 15 min and washed. Colonies containing more than 50 cells were counted using the Image J software and the survival fractions were calculated.

### Cell proliferation

Cell proliferation was measured with eFluor™ 670 (Invitrogen, USA), which is a red fluorescent dye that has a peak excitation of 647 nm. For this assay, 1 × 10^5^ cells were treated with 5 μM eFluor 670, seeded in 12-well plates, then transfected with LZTFL1 siRNAs, miR-21 mimic or miR-21 inhibitor and corresponding controls after 24 h and cultured for another 24 h. Cell proliferation was detected using a flow cytometer (Becton Dickinson, USA) with a 660/20 bandpass filter which is equivalent to allophycocyanin (APC). Mean fluorescence intensity was negative related to cell proliferation rate.

### Wound healing assay

Cells were seeded in 6-well plates and cultured for 12 h. Then a plastic pipette tip was used to produce, and the cells were washed with PBS. The wound closure was observed under a microscope (Olympus, USA) at 0 h and 48 h. Then the relative percentage of the wound closure was calculated.

### Transwell assay

Cells were cultured and then suspended in serum-free culture medium. Then transfer 100 μl of cell suspension to the upper chambers of a Transwell apparatus (Corning, USA). Then the lower chambers were supplemented with 600 μl DMEM containing 10% FBS. The migrating cells to the lower chambers were fixed with methanol and stained with 0.1% crystal violet (Sigma-Aldrich, USA). Migrating cells were counted under a microscope (Olympus, USA). Then the relative migrating ability was calculated.

### Tumor xenograft

Six-week-old BALB/c nude mice were purchased from Beijing HFK Bio-Technology (Beijing, China). The mice were raised and managed at Laboratory Animal Center of HuaZhong University of Science and Technology. The mice were divided into two groups in random. 5 × 10^6^ of control Hs578T cells and miR-21 overexpressing Hs578T cells were injected to each mouse in situ in 100 μl PBS [37]. Tumor volumes were assessed by caliper measurements and calculated as: V = D × d^2^ × 0.5 (D, the longer diameter; d, the shorter diameter). At the end of experiments, mice were euthanized by CO_2_ inhalation. For metastasis, cells were injected into the tail vein. After sacrifice by CO_2_ inhalation, the lung and liver tissues of each mouse were isolated for analysis, based on the approval of the institutional Animal Care and Use Committee of Laboratory Animal Center, HuaZhong University of Science and Technology.

### Immunofluorescence analysis

Cells were seeded in eight-well chamber slides (Millicell EZ SLIDE, Millipore, Darmstadt, Germany) and cultured overnight. After washing with 1 × PBS medium, cells were fixed with pre-cooled methanol for 15 min at − 20 °C. Then the cells were rinsed with 1 × PBS medium, and incubated with 0.2% Triton in PBS for 10 min at room temperature. Cells were then blocked in 5% donkey serum for 45 min at room temperature. After incubation with primary antibody in 1 × PBS containing 1% BSA at 4 °C overnight, the cells were washed with 1 × PBS and incubated with a secondary antibody in 1 × PBS containing 1% BSA for 45 min. Then cells were washed and stained with DAPI for 10 min and observed by a fluorescence microscope (Olympus BX53, Japan). Colocalization rate was calculated by Image J software.

### Immunohistochemistry

Formalin-fixed, paraffin-embedded tissues were obtained from nude mice. Tissues were sliced into ~ 5-μm sections and stained with antibodies. IHC was performed as previously described [37]. Staining intensity and proportion were viewed and expression scores were calculated [38].

### Bioinformatics and statistical analysis

Target genes of miR-21 were predicted by MIRDB (http://mirdb.org/), TargetScan 5.1 (http://www.targetscan.org/), PicTar (http://pictar.mdc-berlin.de/), and miRanda (http://www.microrna.org/). K-M Plot software (http://www.kmplot.com/breast) was used to evaluate survival analysis between LZTFL1 expression level and breast cancer. SPSS19.0 software (Chicago, USA) was used for statistical analyses. Differences between groups were evaluated using a two-tailed Student’s t-test or one-way analysis of variance (ANOVA). *p* values < 0.05 were considered to be significant.

## Results

### Plasma miR-21 level is upregulated in breast cancer

**Fig. 1:**
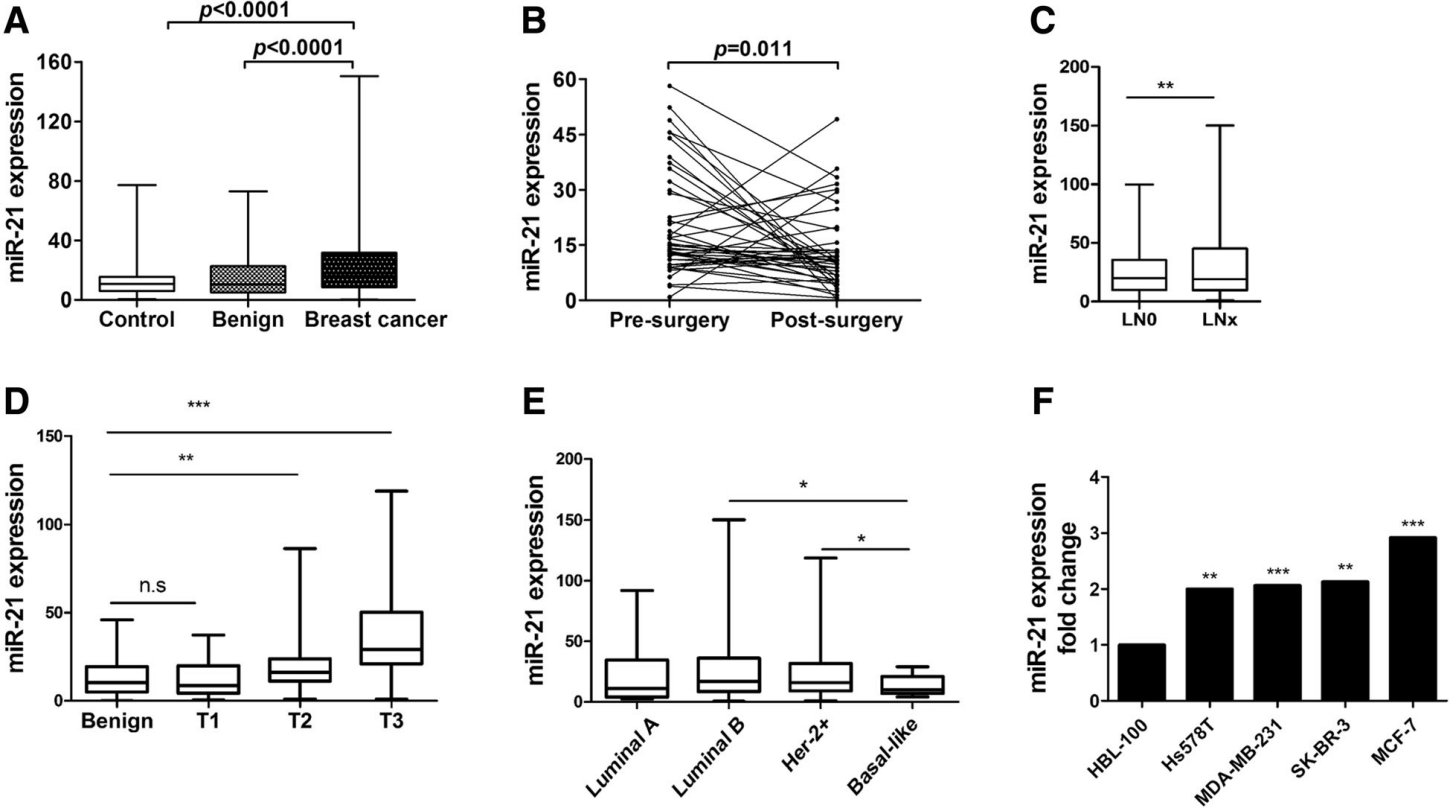
The levels of miR-21 in breast cancer patient plasma and cell lines**. a** Plasma miR-21 levels in 252 breast cancer patients, 127 healthy controls, and 82 benign breast cancer patients (*p* < 0.0001). **b** Plasma miR-21 levels of 44 paired plasma samples from breast cancer patients before and after surgery (*p* < 0.05). **c** Plasma miR-21 levels in the lymph nodes of metastasis-negative (LN0) or -positive (LNx) breast cancer patients (*p* < 0.01). **d** Plasma miR-21 levels in benign breast cancer and stage T1, T2, and T3 breast cancer patients (*p* < 0.01). **e** Plasma miR-21 levels in luminal A, luminal B, Her-2^+^ and basal-like types of breast cancer patients (*p* < 0.05). **f** The mRNA levels of miR-21 in HBL-100, Hs578T, MDA-MB-231, SK-BR3, and MCF-7 cell lines (*p < 0.05, **p < 0.01, ***p < 0.001)

To investigate the plasma level of miR-21 in breast cancer patients, we performed RT-PCR on plasma samples from a large cohort of first diagnostic breast cancer patient. The clinical characteristics of these patients are listed in Table 1. Plasma miR-21 levels were significant higher in 252 breast cancer patients compared with either 127 healthy controls or 82 benign breast cancer patients (Fig. 1a). Importantly, the plasma levels of miR-21 were significantly decreased after surgery comparing with pre operation in 44 patients (Fig. 1b). Moreover, by analyzing the differences between plasma miR-21 with different breast cancer stages T1, T2, and T3, as well as with the different clinical histopathological features, samples from lymph node metastatic breast cancers showed signifacanted upregulation of miR-21 (Fig. 1c). Compared with benign breast cancer samples, plasma miR-21 levels were also elevated in developed breast cancer stages (T2 and T3, Fig. 1d). Taking into account the histopathological features of clinical samples, plasma miR-21 levels were upregulated in luminal B and Her-2^+^ types of breast cancers compared with luminal A and basal-like types, which indicates that miR-21 levels might be related to estrogen receptor (ER) and Her-2 status in breast cancer (Fig. 1e). In addition, by using Oncomine and the Cancer Genome Atlas (TCGA) database, we found that miR-21 mRNA level is higher in invasive breast cancer tissue, compared with its level in normal breast tissues, and high level of miR-21 is related to poor outcome for breast cancer patients (Additional file 1: Figure S1). Next by confirming the expression of miR-21 in vitro*,* we checked its expression from cultured breast cancer cell lines and found that miR-21 was increased in breast cancer cells compared with the immortalized mammary epithelial cell line HBL-100 (Fig. 1f). These results are evidence that miR-21 levels are upregulated in breast cancer and play a key role in the progression of breast cancer.

**Table 1:** The relationship between miR-21 levels and clinicopathological characteristics of breast cancer patients

Patients frequency(%)		miR-21 expression		*P* -value
		Low expression	High expression	
	Total *N* = 252	*N* = 126	*N* = 126	
Age (years)				
≤ 50	75 (29.7%)	35 (27.8%)	40 (31.7%)	0.086
> 50	177 (70.3%)	91 (72.2%)	86 (68.3%)	
Tumor size (cm)				
≤ 3.0	142 (56.3%)	82 (65%)	60 (47.6%)	0.002
> 3.0	100 (43.7%)	44 (35%)	66 (52.4%)	
LN metastasis				
Negative	103 (41%)	63 (50%)	40 (31.7%)	0.001
Positive	149 (59%)	63 (50%)	86 (68.3%)	
TNM stage				
I	51 (20.2%)	46 (36.5%)	5 (4%)	I*vs*II < 0.005
II	11 (46.8%)	63 (50%)	55 (43.6%)	II*vs*III < 0.001
III	83 (33%)	17 (13.5%)	66 (40%)	I*vs*III < 0.001
PR expression				
Negative	66 (26.2%)	30 (23.8%)	36 (28.6%)	0.07
Positive	186 (73.8%)	96 (76.2%)	90 (71.4%)	
ER expression				
Negative	68 (27%)	31 (24.6%)	37 (29.4%)	0.07
Positive	184 (73%)	95 (75.3%)	89 (70.6%)	
HER-2 expression				
Negative	78 (31%)	44 (34.9%)	34 (27%)	0.029
Positive	174 (69%)	82 (65.1%)	92 (73%)	

Low expression and high expression of miR-21 was determined by the cut-off values (18.6) for miR-21, which were defined as the cohort median

### Inhibition of miR-21 reduced breast cancer proliferation and metastasis

**Fig. 2:**
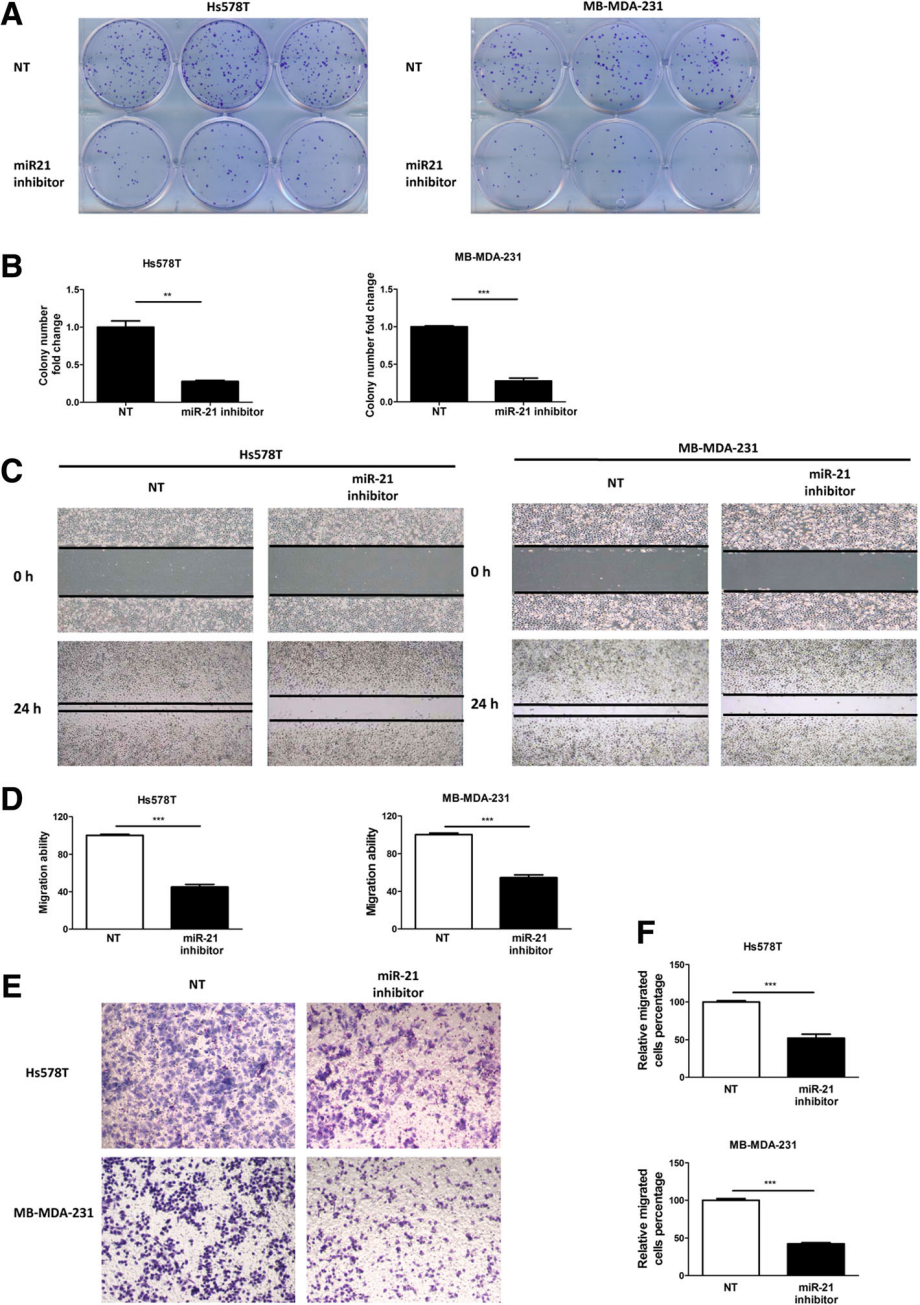
Inhibition of miR-21 reduced colony formation and cell migration in breast cancer. **a** Colony-formation assay for Hs578T and MDA-MB-231 cells transfected with miR-21 inhibitor or non-targeted control (NT). The number of colonies was determined after 14 days. **b** Fold changes in colony number in the absence and presence of inhibitor were compared in three independent experiments. **c** Wound healing assay in Hs578T and MDA-MB-231 cells transfected with miR-21 inhibitor (40× magnification). **d** The relative wound closure was calculated after the experiments were performed in triplicate. **e** Transwell assay in Hs578T and MDA-MB-231 cells transfected with miR-21 inhibitor (100× magnification). **f** The relative migration of cells was determined after the experiments were performed in triplicate (*p < 0.05, **p < 0.01, ***p < 0.001)

To investigate the biological function of miR-21, we performed colony formation, wound healing, and Transwell assays following miR-21 inhibition in Hs578T and MB-MDA-231 cells. It was found that colony formation by breast cancer cells was reduced following treatment with miR-21 inhibitor (Fig. 2a, b). These results indicate that miR-21 maintains breast cancer cell growth. The capacity for cell migration was determined using wound healing and Transwell assays. The results show that wound closure was reduced in breast cancer cells treated with a miR-21 inhibitor (Fig. 2c, d). Similar results were also observed in Transwell assays (Fig. 2e, f). Overall, these results indicate that inhibition of miR-21 reduces breast cancer proliferation and metastasis.

### LZTFL1 is a direct target of miR-21

**Fig. 3:**
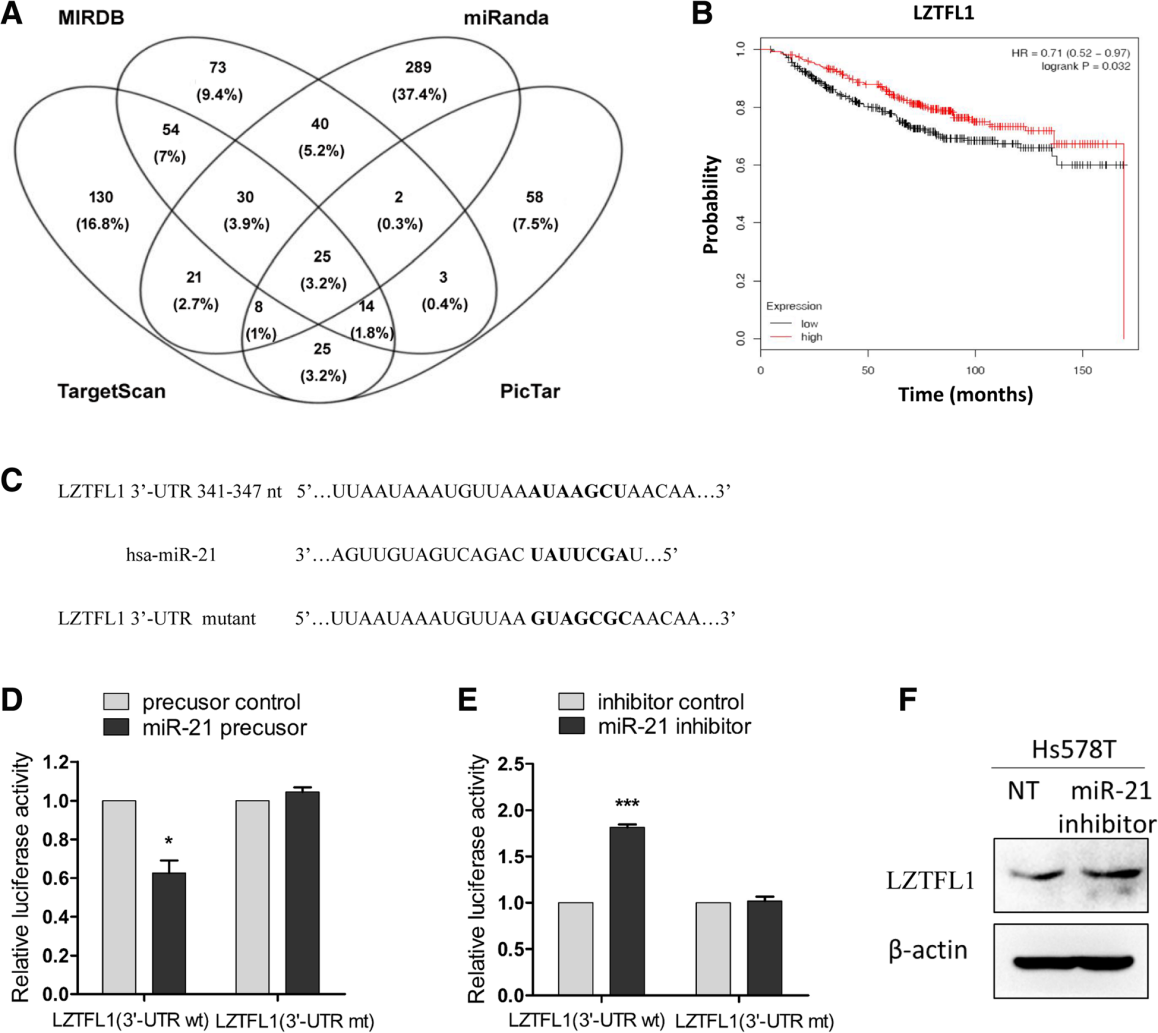
LZTFL1 is a target gene of miR-21**. a** Screening for miR-21 target genes by prediction softwares MIRDB, TargetScan, PicTar, and miRanda. **b** The overall survival rates of breast cancer patients with low (*n* = 319) or high (*n* = 307) expression levels of LZTFL1 were estimated with the Kaplan–Meier method by log-rank test according to data from the Cancer Genome Atlas (TCGA). **c** The predicted binding site of miR-21 in the 3′-UTR of wild type and mutant LZTFL1. **d** and **e** HEK-293 T cells were co-transfected with pRL-TK carrying a wild-type or mutant 3′-UTR sequence of LZTFL1 and the miR-21 precursor (60 ng) or the miR-21 inhibitor (10 pmol), and the luciferase activity was measured at 48 h. Experiments were performed in triplicate. **f** The protein level of LZTFL1 in Hs578T cells after transfection with miR-21 inhibitor

Genes targeted by miR-21 were screened using prediction software, including MIRDB, TargetScan, PicTar and miRanda (Fig. 3a). Among the genes common to all four databases, we chose LZTFL1, a key tumor suppressor, as a target for further research because of its vital role in cancer metastasis. According to the breast cancer data acquired with the TCGA platform, we found that lower expression of LZTFL1 is related to shorter overall survival in breast cancer (Fig. 3b). To validate this potential association, we performed luciferase assays in HEK293T cells using either the wild-type 3′-UTR or the mutant 3′-UTR lacking the miR-21 binding site (Fig. 3c). When miR-21 was overexpressed, luciferase activity was significantly reduced in cells transfected with the luciferase gene with the wild-type 3′UTR of LZTFL1, but not in those with the mutant 3′-UTR (Fig. 3d). Conversely, when miR-21 was inhibited, luciferase activity was significantly increased in cells with wild-type 3′-UTR of LZTFL1, but not in cells with the mutant 3′-UTR (Fig. 3e). Next, we found that expression of LZTFL1 was upregulated in Hs578T cells following miR-21 inhibition (Fig. 3e). These results indicate that LZTFL1 is a direct target of miR-21 in breast cancer.

### The miR-21/LZTFL1 axis promotes cell proliferation and metastasis

**Fig. 4:**
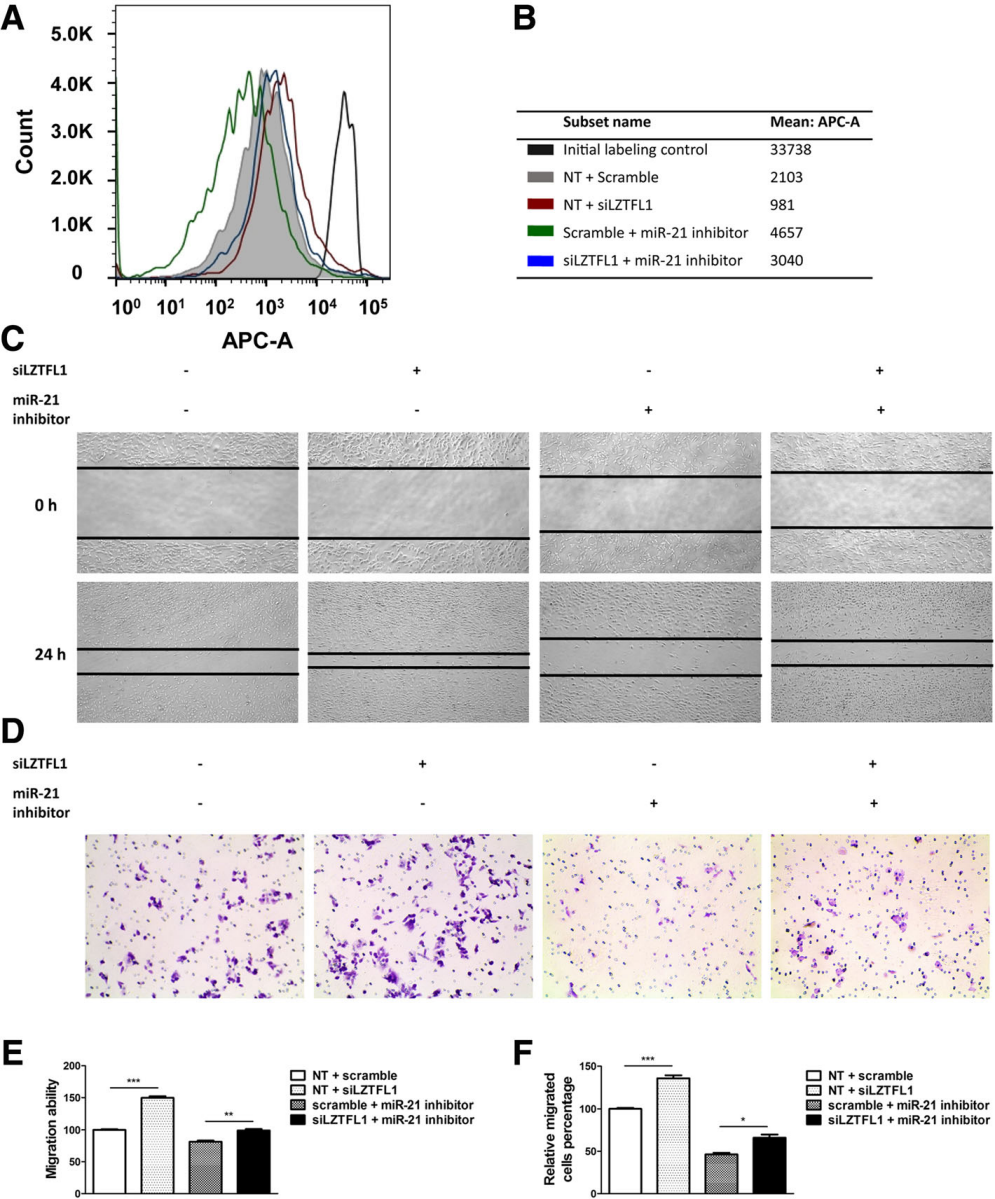
LZTFL1 knockdown reverses miR-21 inhibitor-induced suppression of breast cancer proliferation and migration. **a** The original parental Hs578T cells (0 h) was labeled with eFluor™ 670 dye represented as the initial labeling control group. Then the labeled cells were treated with miR-21 inhibitor, LZTFL1 siRNA alone, or combined for 48 h represented as dividing shifted populations. **b** The mean fluorescence value of each group were caculated. **c** Wound healing assays in Hs578T cells treated with miR-21 inhibitor, LZTFL1 siRNA alone, or combined (40× magnification). **d** Transwell assay in Hs578T cells following the treatments indicated above (100× magnification). **e** Relative wound closure was calculated for the data in (**c**), and the experiments were performed in triplicate. **f** The relative percentage of migrated cells was determined for the data in (**d**), and the experiments were performed in triplicate. (*p < 0.05, **p < 0.01, ***p < 0.001)

Cell proliferation was estimated using the dye eFluor 670, which binds to any cellular protein containing primary amines and is distributed equally between daughter cells as the cells divide. The initial labeling was used as a positive control indicating the fluorescence of the first generation. The results showed that cell proliferation was reduced in Hs578T cells treated with an miR-21 inhibitor, while cell proliferation was increased following the knockdown of LZTFL1 (Fig. 4a, b). Furthermore, knockdown of LZTFL1 overcame the suppressive effect of miR-21 inhibitor on cell proliferation. In addition, we found that LZTFL1 knockdown promoted cell migration in Hs578T cells according to wound healing and Transwell assays (Fig. 4c–f), although it also reversed the effects of the miR-21 inhibitor. These results indicate that *LZTFL1* is a target gene of miR-21 that functions in the process of regulating breast cancer cell proliferation and metastasis. These results demonstrate that miR-21/LZTFL1 promotes breast cancer proliferation and metastasis in vitro.

### The miR-21/LZTFL1/β-catenin axis promotes EMT

**Fig. 5:**
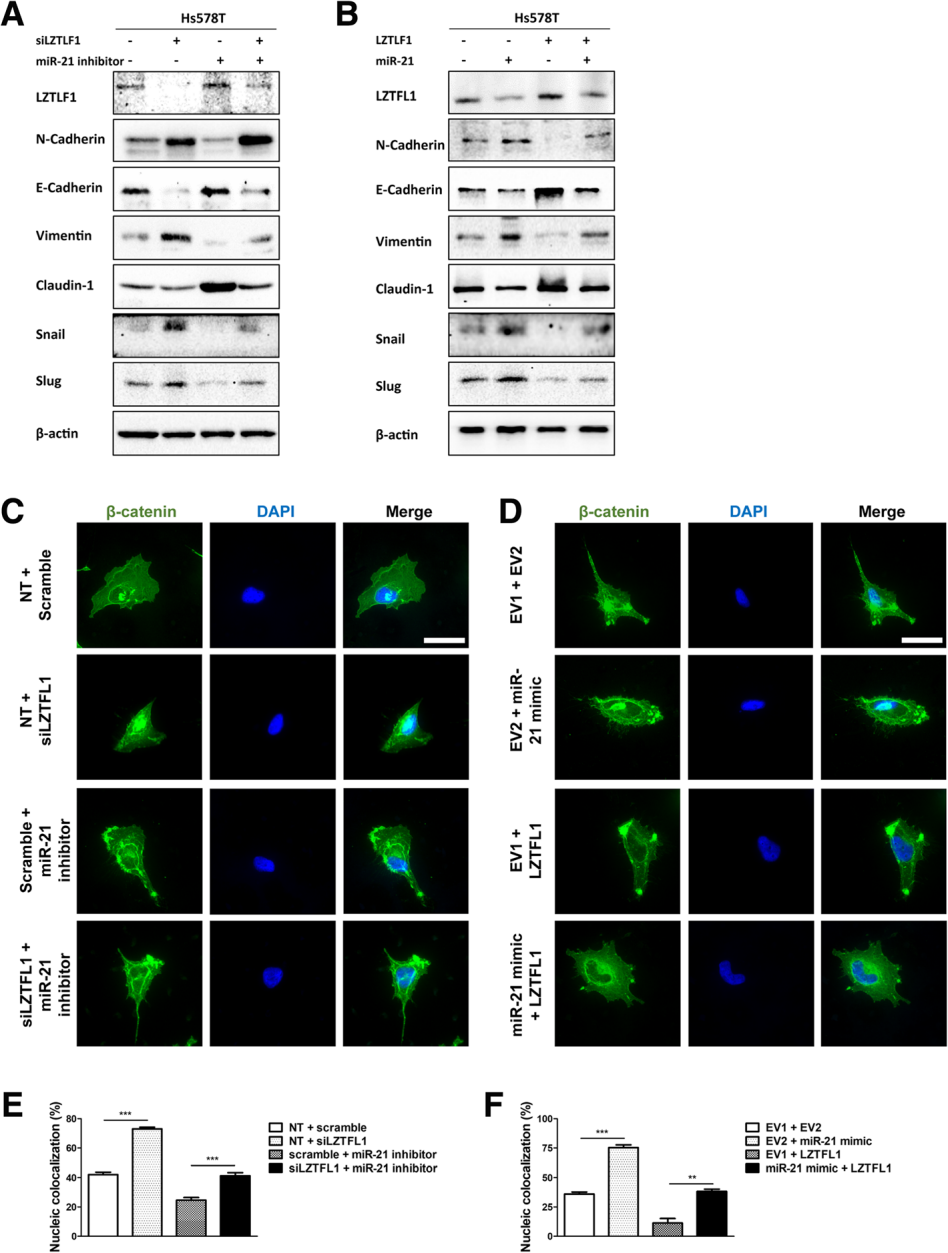
miR-21/LZTFL1 regulates β-catenin nuclear translocation and EMT process. **a** The protein levels of EMT markers in Hs578T cells treated with miR-21 inhibitor, LZTFL1 siRNA alone, or combined for 48 h. **b** The protein levels of EMT markers in Hs578T cells treated with miR-21 mimic, LZTFL1 overexpressing plasmid alone, or combined for 48 h. **c** and **d** Immunofluorescence microscopy analysis of β-catenin nuclear translocation in Hs578T cells following the treatments indicated in (**a**) and (**b**). **e** and **f**. Colocalization rate representing relativeβ-catenin nuclear translocation in (**c**) and (**d**). (*p < 0.05, **p < 0.01, ***p < 0.001)

Since the EMT is a crucial mechanism in tumor metastasis, we next speculated that the miR-21/LZTFL1 axis is involved in the EMT. We detected the protein levels of several EMT markers. The results showed that the protein level of N-cadherin and vimentin were reduced, the levels of E-cadherin and claudin-1 were increased in Hs578T cells following miR-21 inhibition (Fig. 5a). Whereas, the N-cadherin and vimentin levels were increased, the E-cadherin and claudin-1 levels were decreased in LZTFL1 knockdown cells. Moreover, knocking down LZTFL1 restored the suppressive effects on EMT caused by miR-21 inhibitor. In addition, LZTFL1 overexpression also blockade the positive effects on EMT mediated by miR-21 mimic (Fig. 5b). Previous researches reported that LZTFL1 could bind and suppress β-catenin nuclear translocation, and the EMT-promoting transcription factors snail and slug were directly or indirectly regulated by β-catenin [33–35]. Next, we used immunofluorescence assay and detected the nucleic location of β-catenin after the treatment of LZTFL1 siRNA, miR-21 inhibitor, or LZTFL1 overexpressing plasmid and miR-21 mimic. Be consistent with previous study, we found that LZTFL1 suppressed the nuclear translocation of β-catenin (Fig. 5c-f). We also observed that miR-21 promoted the nucleic colocalization of β-catenin. Disruption of LZTFL1 expression could overcome the effects of miR-21 on β-catenin. Besides, snail and slug levels were positive related to the nucleic colocalization rate of β-catenin (Fig. 5a-b). Together, these results implicated that miR-21/LZTFL1 axis might promote breast cancer EMT via β-catenin.

### miR-21 promotes breast cancer proliferation and metastasis in vivo

**Fig. 6:**
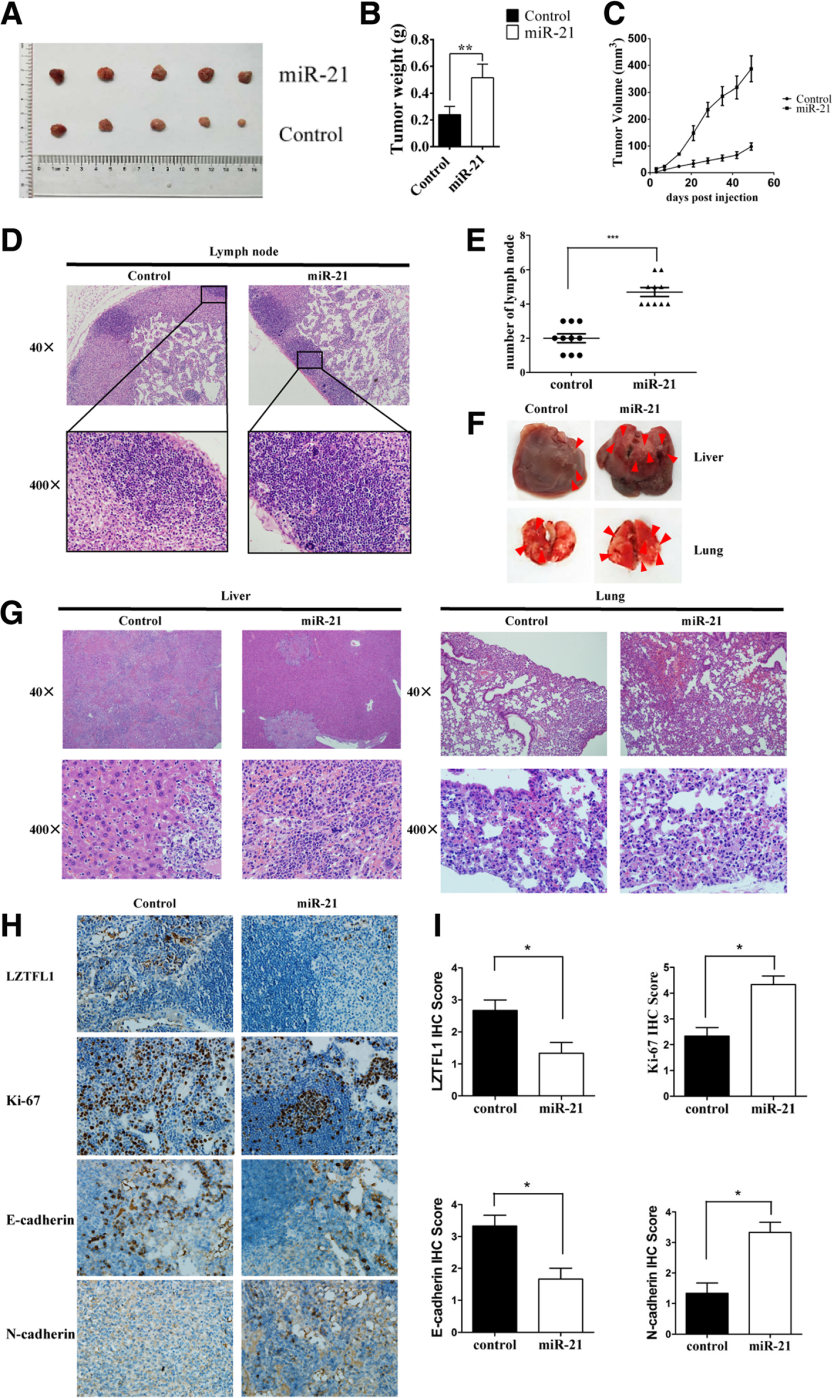
miR-21 promotes breast cancer proliferation and metastasis in vivo. **a** Xenografted tumors were obtained from miR-21-treated Hs578T and control Hs578T cells in situ. **b** and **c** Tumor weight and volume were observed and recorded in the groups indicated above. **d** and **e** The number of lymph nodes invaded was determined. **f** and **g**. Liver and lung tissues were obtained, and the metastatic cells were visualized. **h** and **i**. Immunohistochemistry analysis of LZTFL1, Ki-67, E-cadherin, and N-cadherin in xenografted tumors

To determine the role of miR-21 in breast cancer progression in vivo, BALB/c nude mice were injected with Hs578T cells in situ, and stable overexpression of miR-21 was obtained. We found that miR-21 overexpression significantly promoted tumor growth in vivo (Fig. 6a–c). The number of lymph nodes invaded, which was determined in order to measure the extent of metastasis, was increased in the miR-21 overexpression group (Fig. 6d, e). To further study tumor metastasis, BALB/c nude mice were treated with Hs578T cells using tail vein injection. Liver and lung samples were obtained to evaluate the propensity for tumor metastasis, and the results showed that metastatic cells in liver and lung from these mice were increased in the miR-21 overexpression group (Fig. 6f, g). Moreover, the protein expression level of LZTFL1 was significantly decreased in tumor tissues with miR-21 overexpression (Fig. 6h, i). Meanwhile, the expression of Ki-67 and N-cadherin was increased, while E-cadherin was decreased by miR-21 overexpression. These results suggest that miR-21 promotes tumor growth and metastasis by activating the EMT process in breast cancer (*p < 0.05, **p < 0.01, ***p < 0.001).

## Discussion

Although studies have already revealed the importance of miR-21 as an oncogene, its new target genes, precise molecular mechanisms and clinical potential are still needed further exploration [13–21]. In order to confirm the clinical value of miR-21 in breast cancer, we detected the plasma miR-21 levels in several groups of patients. We found that plasma miR-21 levels were significant higher in breast cancer patients’ samples, compared with healthy controls and benign breast cancer patients’ samples. These findings were also approved by other studies [39, 40]. Moreover, plasma miR-21 levels of breast cancer patients tended to decline following surgery, and plasma miR-21 levels were correlated with lymph node metastasis and the TNM stage in breast cancer. According to these results, plasma miR-21 could be a promising biomarker in the diagnose and outcome prediction of breast cancer. In this sense, its novel targets and mechanisms involved in breast cancer metastasis need to be discovered.

In our study, *LZTFL1*, a new target gene of miR-21, was identified by a screen based on four prediction databases. Then we confirmed the regulation of miR-21 on LZTFL1 by luciferase reporter and western blot assays. Studies have shown that LZTFL1 is significantly downregulated in several type of cancers, which associated with shorter overall survival of patients [32, 33]. Analysis based on TCGA database also suggests that low expression of LZTFL1 predicts a poor outcome in breast cancer. According to these data, we selected LZTFL1 as novel target of miR-21 for further investigation. We confirmed that miR-21 promotes cell proliferation, metastasis, and tumor progression in breast cancer, while knockdown of LZTFL1 reverses these effects. Therefore, the function of miR-21 in promoting breast cancer progress is due, in significant part, to its suppression on LZTFL1.

**Fig. 7:**
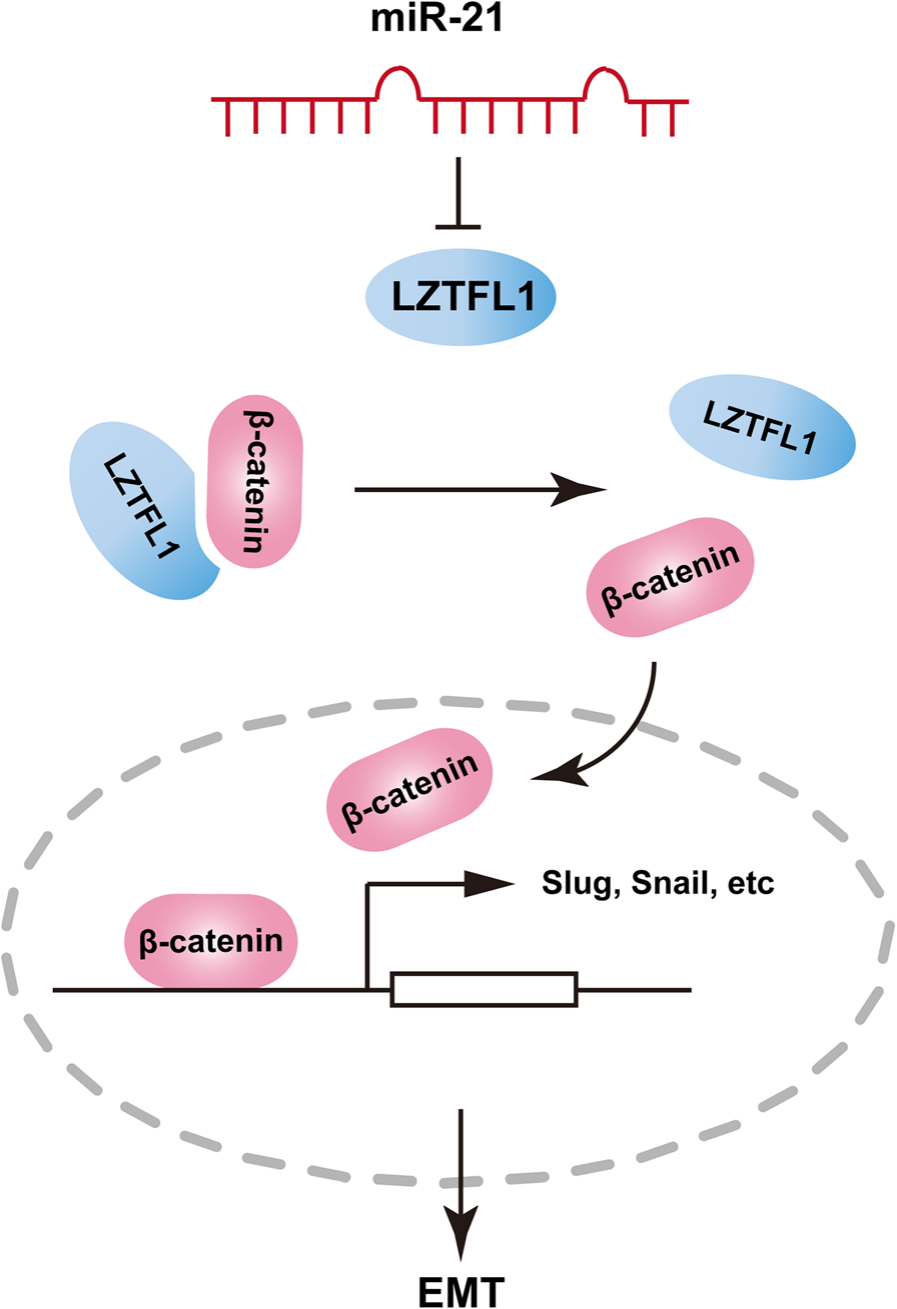
A schematic diagram of miR-21/LZTFL1/β-catenin/EMT axis mediated tumor metastasis in breast cancer. LZTFL1 was suppressed by miR-21 in breast cancer, which increases the nuclear translocation of β-catenin and promotes the transcription of EMT makers

However, the mechanisms of the miR-21/LZTFL1 axis in regulating breast cancer metastasis remain to be determined. Research indicates that LZTFL1 plays a vital role in regulating the EMT process in several cancers [34]. It inhibits tumorigenesis by stabilizing E-cadherin-mediated adherens junction formation in HeLa cells and suppresses gastric cancer metastasis by preventing nuclear translocation of β-catenin [33–35]. It was also reported to inhibit mitogen-activated protein kinase (MAPK) signaling, which decreases the EMT in lung cancer [33]. In our study, we also found that LZTFL1 prevents breast cancer progression by inhibition of the EMT. Since N-cadherin, E-cadherin, vimentin and claudin-1 are EMT associated markers [41–43], we found that disruption of LZTFL1 abrogated the effects of miR-21 on the expression of these markers. To further explore the mechanism underlying this process, we analyzed several signal pathways which could regulate EMT in our model. We observed that miR-21/LZTFL1 regulates the nuclear translocation of β-catenin and its downstream transcription factors snail and slug (Fig. 7). Furthermore, an in vivo study also demonstrated that the miR-21/LZTFL1 axis regulates the EMT to promote metastasis in breast cancer. The expression of LZTFL1 and E-cadherin were decreased after miR-21 overexpression, while the expression of Ki-67 and N-cadherin were increased following treatment.

In conclusion, our results suggest that the miR-21/LZTFL1/β-catenin/EMT axis promotes metastasis via EMT process in breast cancer. Therapies that result in re-expression of LZTFL1 or inhibition of miR-21 might be promising new approaches to targeted therapy for breast cancer.

## Conclusions

This study indicate that plasma miR-21 level is a crucial biomarker for breast cancer diagnosis. LZTFL1 is identified as a novel target of miR-21. Down-regulation of miR-21 inhibits breast cancer cell proliferation and EMT-mediated metastasis in vitro and in vivo by promoting LZTFL1 expression. Mechanically, miR-21/LZTFL1 axis promotes the nuclear translocation of β-catenin which actives EMT process in breast cancer. We describe a specific mechanism that explains the effects of miR-21 in breast cancer. Targeting miR-21/LZTFL1/β-catenin/EMT axis might be a promising strategy in breast cancer therapy.

## Additional file

Additional file 1: **Figure S1.** The expression level and survival analysis of miR-21 in breast cancer patients from TCGA database. A. The overall survival rates of breast cancer patients (*n* = 1061) with low or high expression levels of miR-21 were estimated with the Kaplan–Meier method by log-rank test according to data from the Cancer Genome Atlas (TCGA) on Kaplan–Meier Plotter platform (http://kmplot.com/analysis/). B. The relative miR-21 expression levels in normal breast tissue (*n* = 61) and invasive breast cancer tissue (*n* = 76) were analyzed according to data from TCGA provided by Oncomine (https://www.oncomine.org). (PDF 189 kb)

## Data Availability

All data generated or analyzed during this study are included in this published article.

## Abbreviations

APC: Allophycocyanin
EMT: Epithelial-to-mesenchymal transition
ER: Estrogen receptor
GADPH: Glyceraldehyde-3-phosphate dehydrogenase
LZTFL1: Leucine zipper transcription factor-like 1
MAPK: Mitogen-activated protein kinase
miRNAs: microRNAs
PAGE: Polyacrylamide gel electrophoresis
SDS: Sodium dodecyl sulfate
UTR: Untranslated region
