# Supplementary material for: microRNA-21 promotes breast cancer proliferation and metastasis by targeting LZTFL1
Source: BMC Cancer. 2019 Jul 27;19:738. doi: 10.1186/s12885-019-5951-3 (PMC6661096; doi:10.1186/s12885-019-5951-3)
Supplement: Supplementary file 1 — Figure S1. The expression level and survival analysis of miR-21 in breast cancer patients from TCGA database. A. The overall survival rates of breast cancer patients (n = 1061) with low or high expression levels of miR-21 were estimated with the Kaplan–Meier method by log-rank test according to data from the Cancer Genome Atlas (TCGA) on Kaplan–Meier Plotter platform (http://kmplot.com/analysis/). B. The relative miR-21 expression levels in normal breast tissue (n = 61) and invasive breast cancer tissue (n = 76) were analyzed according to data from TCGA provided by Oncomine (https://www.oncomine.org). (PDF 189 kb) [file 12885_2019_5951_MOESM1_ESM.pdf]

Supplemental Materials

1. Supplementary Figure

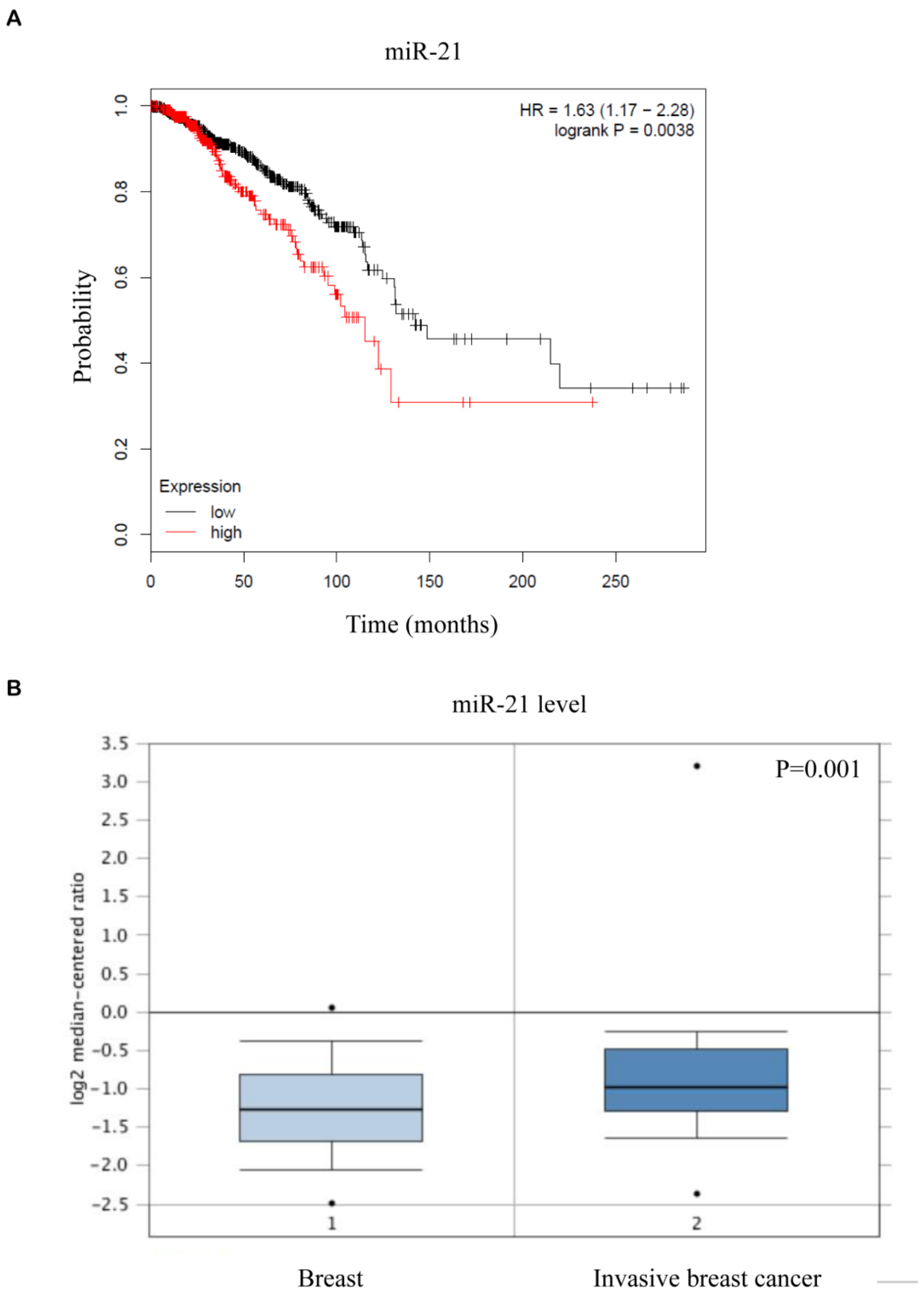

Figure S1. The expression level and survival analysis of miR-21 in breast cancer patients from TCGA database. A. The overall survival rates of breast cancer patients (n=1061) with low or high expression levels of miR-21 were estimated with the Kaplan–Meier method by log-rank test according to data from the Cancer Genome Atlas (TCGA) on Kaplan–Meier Plotter platform (http://kmplot.com/analysis/). B. The relative miR-21 expression levels in normal breast tissue (n=61) and invasive breast cancer tissue (n=76) were analyzed according to data from TCGA provided by Oncomine (https://www.oncomine.org).
